# Anatomical landmarks in auditory brainstem implant surgery

**DOI:** 10.1016/S1808-8694(15)31324-0

**Published:** 2015-10-20

**Authors:** Rubens Vuono Brito Neto, Ricardo Ferreira Bento, Alexandre Yasuda, Guilherme Carvalhal Ribas, Aldo Junqueira Rodrigues

**Affiliations:** ^1^ Ph.D., Assistant physician; ^2^ Associate Professor, Discipline of Otorhinolaryngology, FMUSP; ^3^ Ph.D. in Medical Sciences, Assistant Physician; ^4^ Professor of Neuroanatomy, Discipline of Human Structural Topography, FMUSP; ^5^ Faculty Professor, Discipline Human Structural Topography, FMUSP. HC-FMUSP

**Keywords:** cadaver, dissection, dye dilution technique, auditory brainstem implant/education, cochlear implantation/trends

## Abstract

**A**uditory brainstem implant (ABI) is an option for deaf patients who do not have the whole auditory pathways preserved. The surgery, because of its anatomical and functional complexity, requires specific training of the surgeon in an anatomy lab. **Aim**: To study the surgical anatomy of the auditory brainstem implant surgery. **Study design**: Anatomic study. **Material and Method**: In the present study, we dissected a fresh cadaver prepared with a dye solution injected into the arteries and intracranial veins. The location of the insertion of the ABI electrode was studied through translabyrinthine access. **Results**: The surgical technique used for implanting the brainstem electrode is similar to that used in the removal of vestibular schwannoma. The cochlear nucleus complex, comprising ventral and dorsal cochlear nuclei, is the optimal electrode site. The ventral cochlear nucleus is the principal nucleus for transmission of neural impulses from the 8th pair and form the main ascending route of the cochlear nerve. Neither the ventral nor the dorsal nuclei are visible during surgery and their location depends on the identification of adjacent anatomical structures. **Conclusion**: The region for the implantation of the electrode in the auditory brainstem implant presents anatomical landmarks that allow its easy identification during surgery.

## INTRODUCTION

Auditory brainstem implant was developed to restore some useful hearing in patients that presented absence of bilateral cochlear nerve. It was primarily developed as a monochannel electrode at House Ear Institute (HEI), in Los Angeles, California. This first model was used in 25 patients between 1979 and 1992 with poor clinical results^1^. Based on this experience, they developed a multichannel implant together with HEI and Cochlear Corporation (Englewood, Colorado) and Huntington Medical Research Institute (Pasadena, California). Even though the first surgery for implantation of auditory brainstem implant dated back from 1979, only in October 2000 there was approval to its clinical use^2^.

The patients that are classically benefited from this type of surgically implantable electronic hearing aid are those with diagnosis of type 2 neurofibromatosis (NF-2) because they present bilateral vestibular schwannomas or children with cochlear nerve congenital aplasia. Currently, the indication of auditory brainstem implant has expanded and patients with neural integrity of 8th nerve and impossibility of having a conventional cochlear implant, such as people with ossified cochleae after meningitis, are potential candidates to this surgery3., 4., 5.. This fact is important because despite the fact that indication criteria for cochlear implant have expanded quite a lot in past years, it may not be used in the above mentioned cases6., 7., 8.. There are patients that do not benefit from this technology because they do not receive electrical stimulation of peripheral auditory pathways (cochlea and spiral ganglion). In our list of patients waiting for surgery for bilateral vestibular shwannomas (type 2 neurofibromatosis), at least eight subjects could have immediately benefited from brainstem implant and there are at least six new patients with this diagnosis with year. Our surgical experience with lateral skull base tumors and the fact that we perform routine vestibular shwannoma surgeries through the universal healthcare system (Sistema Único de Saúde) make us want to provide the service to these patients as well.

In addition to these cases of tumors, other patients with cochlear obliteration after meningitis, which unfortunately form most of our cases in the ambulatory, plus the rare cases of central neuropathy, may benefit from auditory brainstem implant. The next step of our Cochlear Implant Group is to master the surgical technique and to make it available to our patients. In Brazil, not one single patient has been implanted with this type of prosthesis.

Our objective in the present study was to analyze anatomical parameters of the region where the electrode for the auditory brainstem implant is inserted.

## MATERIAL AND METHOD

The present study was developed by laboratory B1, Discipline of General Surgery and Human Structural Topography, Department of Surgery, FMUSP, and Laboratory of Medical Investigation - Otorhinolaryngology (LIM-32), being approved by the Ethics Committee for Analysis of Research Project, Clinical Director's Office, Hospital das Clínicas and Medical School, University of Sao Paulo (Protocol 767/04).

A fresh cadaver was prepared for dissection with injection of dye solution dissolved in liquid silicone. The structure of the dissecting bench was based on the one used at the Hands-on Course of Temporal Bone Dissection, Discipline of Otorhinolaryngology, FM-USP.

The head of the cadaver was stored in alcohol at 75% and prepared for dissection to highlight intracranial vascularization. Internal carotid artery and bilateral internal jugular vein were identified and dissected at the neck region. Vertebral artery was also dissected and isolated bilaterally. A catheter as large as the vessels was introduced through their lumen. Vessels were tied with cotton thread 0 to fix the catheter, preventing liquid reflux. Vessels were repeatedly rinsed with water through a 60cc syringe up to reaching good perfusion of its contralateral correspondence, or in other words, when rising the right internal carotid artery, we expected to have water flowing out of the left internal carotid artery, without presence of blood clots in the vascular lumen. This step was essential because rinsing was insufficient or the presence of clots prevented the input of dyes into the vessels and total obliteration of intracranial vascular system, especially small arterial branches. After the end of rinsing, we injected the dye. Internal and vertebral carotid arteries were injected by the cervical portion and next we injected the internal jugular vein. Arteries were stained in red and the venous system in blue. The preparation of the staining substance followed the formula below:

A. Arteries: two parts of polymethyl silaxane (thinner) to one part of silicone
B. Veins: one part of polymethyl silaxane (thinner) to one part of silicone

Before the injection in the vascular lumen, we added the catalyst (dilaurate calcium carbonate) at a proportion of 10ml for each 300ml of solution. Staining agents (water-soluble pigments) were added right after the infusion.

Anatomy of the region where the auditory brainstem implant is inserted was studied using expanded translabyrinthic access.

## RESULTS

**Figure 1:**
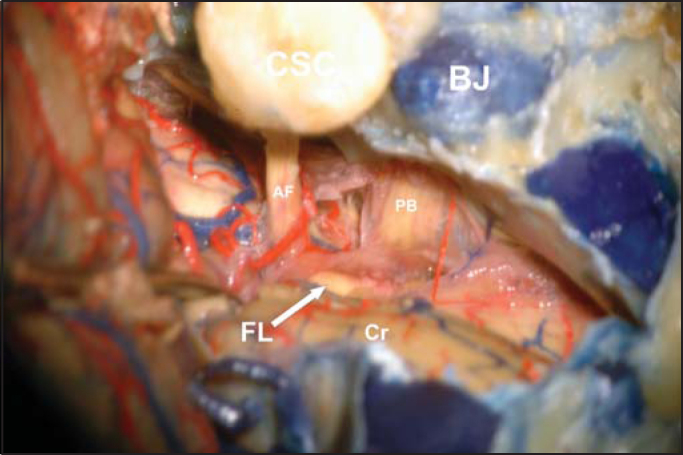
The lateral limits were semicircular canals (SCS) and jugular bulb (BJ), Cranial nerves 7th and 8th (AF), related with the otic capsule, and cranial nerves 9th, 10th and 11th (PB), related to jugular bulb, can be seen from their emergence in the brainstem (cerebellum flocculus moved away with a spatula). Anterior-inferior cerebellar artery goes around the acoustic-facial bundle (AF) in this case. Luschka's foramen (FL) is the white depression on the tip of the arrow (Cr: cerebellum).

**Figure 2:**
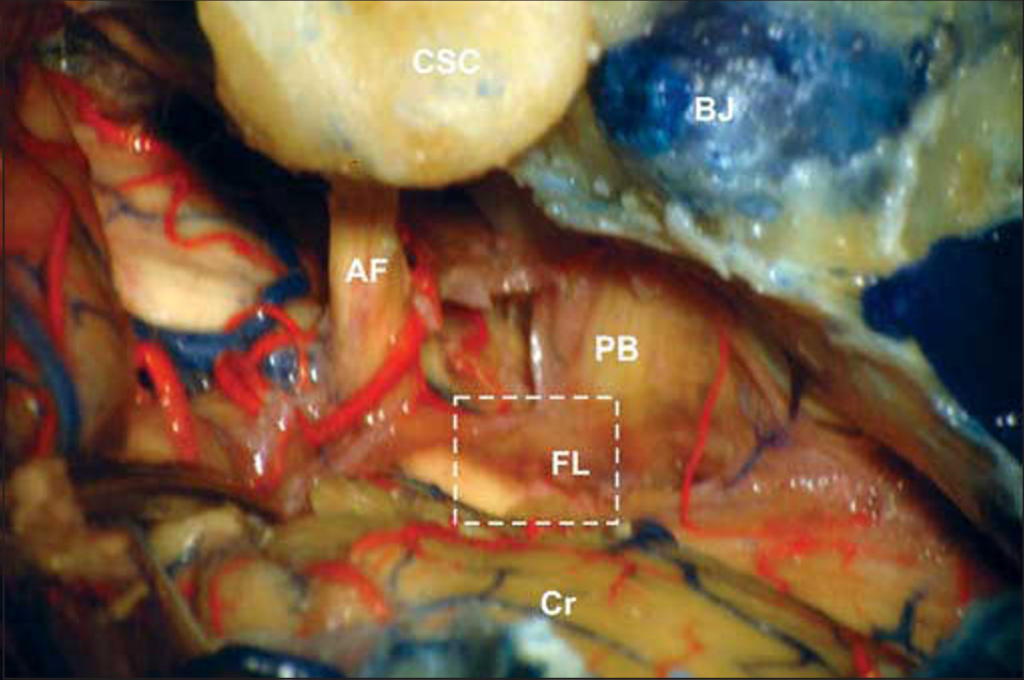
Further magnification showing the depression that exists between the acoustic-facial bundle (AF) and bulb pairs (PB) (white square). This depression corresponds to Luschka's foramen (FL) and it is easily visible after moving away the cerebellum flocculus (BJ: jugular bulb, CSC: semicircular canals, Cr: cerebellum).

The surgical technique for implementation of brainstem electrode is similar to the one used for removal of vestibular shwannoma. Cochlear nucleus complex, comprising the ventral and dorsal cochlear nuclei, is the site for electrode placement. The ventral cochlear nucleus is the main nucleus for transmission of neural impulses from the 8th nerve and its axons form the main ascending tract of the cochlear nerve. Both the ventral and dorsal nuclei are not visible during surgery and their location depends on identification of neighboring anatomical structures. The lateral termination of the fourth ventricle, Luschka's foramen, is placed between the emergence of glossopharyngeal and facial nerves. Getting away from the flocculus, the surgeon can visualize a depression between the above referred cranial nerves, site where the electrode should be inserted (Figures 1 and 2). Normally only a stump of cochlear nerve is identified and can be used as reference for lateral recess.

## DISCUSSION

The concept of auditory brainstem implant is similar to that of cochlear implant currently available, different only regarding the configuration of the electrode, designed to be introduced at the level of the cochlear nerve and not at the tympanic scala of the cochlea. Patients that cannot receive electrical stimuli through the inner ear for anatomical or functional reasons may benefit from this technique. In sociologically advanced countries, the main cause for structural loss of peripheral hearing pathways bilaterally is type 2 neurofibromatosis, whose essential characteristic is to progress with bilateral vestibular shwannomas^9^. However, it does not happen in Brazil. Unfortunately, infectious etiologies are still responsible for most cases of deafness and among them, meningitis is certainly the main one. Based on data still to be published, we observed that 23.9% of all our cases already implanted with some kind of multichannel cochlear implant are patients that have lost their hearing due to meningitis.

This fact is of concern, given that the prognostic of auditory function after implementation is closely related with number of feasible neural elements and to correct positioning of electrodes in the cochlea. Meningitis goes against these two factors. First of all, it is the etiology that causes the most destruction of cochlear hair cells and cochlear nerve neurons, and secondly, it normally takes to some degree of otic capsule ossification. Upon observing our results for sentence discrimination, contained in data to be published, we could see that patients with meningitis had very low discrimination scores (average of 82% in open-ended sentences), results that prevented conversation without support of lip reading. In addition to functional issue, meningitis was responsible for all our six cases of failure in technical positioning of electrode during the implantation surgery, with consequent explanation of the internal unit and need to perform a new surgery to implant new electrodes. This infectious fact expands further the need to have it available as an alternative to conventional cochlear implant in Brazil. Patients with type 2 neurofibromatosis are rare even in a reference center as the one we have at Hospital das Clínicas, FMUSP. There are maximum six cases per year, some without surgical indication. However, taking into account the tragic but slow progression of this disease, the impact of restoring useful hearing to these patients is extremely high. For this reason, we believe that auditory brainstem implant is the next step to be taken by our group of Otology, requiring the study and development of surgical technique at the laboratory of anatomy.

As to the access route used to implant the brainstem electrodes, there are two main options: suboccipital retrosigmoid and translabyrinthic accesses^10^. We believe that the chosen access route should be wide enough to allow correct identification of anatomical parameters used as reference for correct placement of electrodes, and the choice of these two accesses is made according to the experience of the surgeon with each one of them. Most ENT physicians prefer translabyrinthic access in surgeries for removal of large vestibular schwannomas or those with damaged hearing, and thus, implantation is made in the same surgical act, which was also our preferred access. It presents the advantages of identifying the facial nerve before its immersion into the tumor, prevents cerebellum retraction in large tumors and provides direct access to Luschka's foramen. Patients submitted to surgery through this access wake well and quickly, and they rarely have anesthetic problems. The disadvantages are limited exposure to bulb cranial nerves and large vessels of posterior cerebral fossa, which are posterior to the tumor from the surgeons' perspective, and the possibility of anatomical variation of the jugular bulb or sigmoid sinus, or even a small mastoid, which may limit the access. Suboccipital retrosigmoid access is traditionally the preferred one byneurosurgeons. It is quite safe and allows wide exposure of posterior cerebral fossa showing the correlations between the tumor and bulb cranial nerves and large vessels. However, it requires extensive cerebellum retraction, leading to postoperative imbalance and it does not allow early identification of facial nerve. In both accesses, identification of the emergence of the cochlear nerve at the brainstem and coroid plexus should be made clearly marked to act as a reference for electrode positioning^11^.

The electrode insertion site of the auditory brainstem implant is the cochlear nucleus complex, comprising dorsal and ventral nuclei, which corresponds to the site where the cochlear nerve axons end. The dorsal nucleus is located superiorly to lateral recess of 4th ventricle, whereas the ventral nucleus is found recovered by the medium cerebellum peduncle. Therefore, they are not visible to the surgeon and should be located through anatomical references placed on the surface of the pons. Between the emergence of the facial and glossopharyngeal nerves we can find the lateral recess or Luschka's foramen. Dorsal cochlear nucleus is the main nucleus that receive axons from the cochlear nerve and forms the main auditory ascending pathway, but the preferred site for electrode placement is Luschka's foramen, where we can find the junction of ventral cochlear nerve and lower portion of dorsal cochlear nerve, given that this region is the least susceptible to originate non-auditory stimuli, such as facial and glossopharyngeal nerves or adjacent regions such as flocculus and cerebellum^12^. The importance of positioning well the electrode is to prevent side effects of non-auditory neural stimulation. Electrodes positioned at Luschka's foramen have proved to be effective in auditory stimulation with minimum side effects, in addition to proving to be stable at spatially limited sites13., 14..

In our study of surgical dissection we analyzed the references by translabyrinthic access, which we are technically familiar with. There were no difficulties to accurately recognize the lateral recess (Luschka's foramen), a clearly visible depression between the acoustic-facial bundle and bulb cranial nerves. It is necessary to bear in mind that exeresis of large tumors leads to changes in anatomy of the region, especially in relation to emergency of 8th cranial nerve from the pons, difficult to recognize with loss of nerve integrity during surgery or its presence in remains of arachnoid. The observation of anatomically preserved regions in addition to tumor bed, either distal or proximal, and intraoperative electromyography are undoubtedly useful parameters for the procedure of electrode positioning.

## CONCLUSION

The region of electrode implantation in auditory brainstem implant presents anatomical references that allow their identification during the surgery. The study of the surgical technique in the laboratory of anatomy should be encouraged, especially because it is important that surgeons get to know well these landmarks.
